# Therapeutic Strategies for Human Epidermal Receptor-2 Positive Metastatic Breast Cancer: A Literature Review

**DOI:** 10.7759/cureus.9522

**Published:** 2020-08-02

**Authors:** FNU Sapna, Pal Satyajit Singh Athwal, Mukesh Kumar, Sandeep Randhawa, Sukhmanii Kahlon

**Affiliations:** 1 Internal Medicine, Lal Medical Center, Kashmore, PAK; 2 Internal Medicine, Saraswathi Institute of Medical Sciences, Hapur, IND; 3 Neurology, Shaheed Mohtarma Benazir Bhutto Medical University, Larkana, PAK; 4 Internal Medicine, University of Hawaii, Hawaii, USA; 5 Internal Medicine, Medical University of the Americas, Camps, KNA

**Keywords:** her-2 positive breast cancer, breast cancer

## Abstract

Breast cancer is a frequently occurring malignancy in women. Immunologically, breast cancers can be classified into four subtypes depending on the types of receptors present and their expression profiles. These are estrogen positive, progesterone positive, human epidermal growth factor receptor type 2 (HER2) positive, and triple-negative as identified by immunohistochemistry. This classification is the basis of response to treatment, prognosis, and survival. With the identification of HER2 receptor overexpression, targeted therapies with anti-HER2 agents have been developed. The first-line therapy approved for HER2 positive tumors is trastuzumab and pertuzumab linked to taxane and further treatment with an antibody-drug conjugate to achieve satisfactory outcomes. Tyrosine kinase overexpression can be treated with lapatinib, which has also been approved for improving survival and is used in combination with capecitabine. Acquired resistance in HER2 positive tumors is shown in many cases due to genetic or epigenetic modifications. Therefore, it is very important to plan therapeutic strategies and design effective treatment approaches. For a long time, only two agents, trastuzumab and lapatinib, have been approved by the Food and Drug Administration (FDA) for the treatment of HER2 positive breast cancers. There has been no appropriate treatment for trastuzumab resistance and its failure to reduce tumor growth. Lapatinib was approved by the FDA in 2007 for HER2 positive breast cancer. Three existing therapy options after trastuzumab resistance was proposed by clinicians: continuation of trastuzumab, starting therapy with lapatinib, and the synergistic use of trastuzumab and lapatinib.

There have been several effective therapies proposed for HER2 positive breast cancers in correlation with clinical trials. Discovering the mechanisms of trastuzumab resistance would increase its response to therapy and better clinical outcome. Clinicians are being continuously challenged by the resistance mechanisms and bioavailability of the drugs in the treatment of metastatic breast cancers. The addition of new drugs to the chemotherapeutic regimen increases the complexity, burden of side effects, and chances of relapse. Novel anti-HER2 agents have been directed towards therapy making a major paradigm shift.

## Introduction and background

Breast cancer is the most frequently occurring malignancy in women, estimated at about 212,600 cases per year in the United States. It is a major cause of death among women and approximates 15% of deaths in females [1]. Death in breast cancer is due to metastatic disease and systemic involvement. A lot of research has been done in the field of chemotherapy; the odds of achieving complete response is rare. Very few patients have shown complete response after prolonged exposure to chemotherapy. Survivors of metastatic breast cancer either were of younger age or limited metastasis. Response to chemotherapy is initially very rapid but transient, and it fades over a time period of 12-24 months [2]. Various clinical factors are also associated with response to chemotherapy and efficacy in improving survival. Circulating tumor cells (CTCs) found in the blood of patients with metastatic disease can now be detected at very low frequencies by immune magnetic platforms [3]. Some case reports have associated the presence of CTCs with a shorter lifespan in metastatic breast cancer. Treatment planning and early disease detection have the most beneficial effect on the patient’s quality of life. The data concluded that CTCs are important in determining prognostic significance and understanding which patient will benefit from chemotherapy. Hormone receptor-positive tumors with visceral involvement are known to be of somewhat aggressive malignancy and are selected for therapy with cytotoxic agents [4].

## Review

Immunological profile and gene expression in breast cancer

Immunologically, breast cancers can be classified into four subtypes depending on the type of receptors present and their expression profiles. These are estrogen positive, progesterone positive, human epidermal growth factor receptor type 2 (HER2) positive, and triple-negative as identified by immunohistochemistry. This classification is the basis of response to treatment, prognosis, and survival [5]. Triple-negative breast cancers are difficult to treat because they do not have a specific target receptor and worst prognosis. The biological process of tumor growth varies in proliferation, immune response, tumor invasion, vascular growth, apoptosis, receptor signaling, and prognosis. In ER-positive tumors, factors associated with survival are cell proliferation and histologic grading. However, in HER2 positive tumors, immune response, and tumor invasion are the most important factors for prognosis and survival. Triple-negative breast cancers have been found to have a direct association of immune response and lifetime survival. Another study concluded that gene expression is directly proportional to shorter survival in HER2 positive and triple-negative breast cancers. Within the pooled analysis of a study, a total of 996 patients received neoadjuvant chemotherapy, after that gene profiling was done. Gene profiling showed that immunological response was highest in HER2 positive breast cancers and lowest in estrogen receptor-negative cancers [6].

Mechanism of carcinogenesis in HER2 positive breast cancer

Human epidermal growth factor receptor type 2 (HER2) belongs to the tyrosine kinase group of receptors. Overexpression of tyrosine kinase receptor leads to HER2 positive tumors. HER2 produces signal amplification of three transmembrane receptors, which then bind to epidermal growth factors accelerating cell proliferation. HER2 is an oncogene located on chromosome position 17p; it can also be activated by polysomy 17 in some cases [7]. In addition to cell proliferation, it also inhibits apoptosis accounting for the aggressive histopathological picture. The activation of various cell signaling pathways defines its increased chances of metastasis to the brain and resistance development against anti-HER2 agents. It has been reported that non-coding ribonucleic acids (RNAs) and environmental factors are also related to different protein interactions and treatment resistance [8].

Therapeutic management of HER2 positive breast cancers

With the identification of HER2 receptor overexpression, targeted therapies with anti-HER2 agents have been developed. The first-line therapy approved for HER2 positive tumors is trastuzumab and pertuzumab linked to taxane and further treatment with an antibody-drug conjugate to achieve satisfactory outcomes. Tyrosine kinase overexpression can be treated with lapatinib, which has also been approved for improving survival and is used in combination with capecitabine. Acquired resistance in HER2 positive tumors is shown in many cases due to genetic/epigenetic modification. Therefore, it is very important to plan therapeutic strategies and designing effective treatment approaches [9]. The exact mechanism of the action of trastuzumab is not clear yet; different possibilities about its efficacy have been proposed. Trastuzumab acts through antibody-dependent cellular cytotoxicity by the detection of the Fcγ receptor binding to the Fc portion of trastuzumab. This mechanism of action is supported by the mice model exhibiting defective Fcγ receptor was associated with suppressed anti-tumor activity of trastuzumab [10]. Data from a pilot study on 11 patients with HER2 positive tumors showed a positive response to trastuzumab therapy and antibody-dependent cellular cytotoxicity (ADCC) activity [11]. Another mechanism of the action of trastuzumab is by inhibiting HER2 proteolytic cleavage through steric blockage. Some preclinical and clinical studies have found a positive correlation between trastuzumab therapy and serum HER2 levels [12]. Trastuzumab therapy breaks off the PI3K/Akt signaling pathway as stated in two pre-clinical studies. It acts by PTEN upregulation and downregulation of PI3K/Akt function. Few hypotheses have been suggested that trastuzumab acts by causing cell cycle arrest in phase G1 by promoting a cyclin-dependent kinase inhibitor [13]. Cells exhibiting overexpression of HER2 have raised VEGF levels upregulating vascular growth and tumor growth. Trastuzumab acting alone decreasing the activity of vascular endothelial growth factor (VEGF) in HER2 overexpressing cells, whereas, in addition to paclitaxel, it has shown raised intratumoral drug levels and enhanced effects [14]. Sensitivity to trastuzumab depends upon PTEN. Any alterations in the PTEN or PI3K/Akt pathway are the most plausible hypotheses for trastuzumab resistance. Functional RNA interference causes mutations, which result in the activation of PI3KCA and loss of function of PTEN. A retrospective study on 256 patients has shown PIK3CA mutations and PTEN loss associated with trastuzumab resistance [15]. Alteration in IGF1R signaling was the first proposed mechanism found for its resistance. HER2 receptor and trastuzumab binding is sterically inhibited by membrane-associated glycoprotein-4 (MUC-4). As evidence reports, there has been overexpression of membrane-associated glycoprotein-4 (MUC-4) in trastuzumab-resistant cells. Similarly, other preclinical studies demonstrated that trastuzumab resistance was also acquired by overexpression of MUC1 and its action was conversed by MUC1 antagonist [16].

Strategies for trastuzumab-resistant cases of HER2 positive breast cancer

For a long time, only two agents have been FDA approved for the treatment of HER2 positive breast cancers: trastuzumab and lapatinib. There has been no appropriate treatment for trastuzumab resistance and its failure to reduce tumor growth. Lapatinib was approved by the FDA in 2007 for HER2 positive breast cancer. Three existing therapy options after trastuzumab resistance have been proposed by clinicians: continuation of trastuzumab, starting therapy by lapatinib, or synergistic use of trastuzumab and lapatinib [17]. Data from researches have demonstrated that the discontinuation of trastuzumab results in the production of trastuzumab-resistant cell lines. Retrospective data from oncologists suggest that the continuation of trastuzumab with capecitabine was found to be effective in 41% of cases [18]. Lapatinib is a reversible tyrosine kinase inhibitor, and it downregulates the activity of EGFR and HER2 possessing anti-tumor activity [19]. Various preclinical and retrospective studies have shown the activity of lapatinib against HER2 positive trastuzumab-resistant tumors. It is approved for use in patients with prior treatment with taxanes, trastuzumab, and anthracyclines. The synergistic activity of lapatinib with capecitabine is superior to therapy alone with lapatinib [20]. Additionally, lapatinib is also effective in patients with metastasis to the brain in HER2 positive cases, as it can cross the blood-brain barrier easily. Brain metastasis is also frequently found in association with therapy with trastuzumab, as it cannot cross the blood-brain barrier. This highlights the importance of lapatinib and capecitabine in the treatment of HER2 positive breast tumors. In HER2 positive breast cancer, combination therapy of lapatinib and trastuzumab provides dual cell line blockage. Lapatinib enhances the expression of HER2 over the surface of cancer cells and trastuzumab mediates antibody-dependent cytotoxic cell death. A randomized controlled trial of patients treated with trastuzumab and lapatinib both have shown improved clinical outcomes and longer survival. The side effects of dual therapy were increased but its benefits overweigh the side effects [21].

Recent strategies of HER2 positive breast cancers

Recent studies have suggested that antibody and drug conjugates have been found to be associated with decreased HER2 expression. Among these is trastuzumab-deruxtecan. Another combination that proved to be effective is trastuzumab with pembrolizumab [22]. Some researchers have suggested cell cycle inhibitors in combination with trastuzumab as effective management strategies. PATINA (Randomized, Open-Label, Clinical Study of the Targeted Therapy, Palbociclib, to Treat Metastatic Breast Cancer) and MONARCHER (Study of Abemaciclib (LY2835219) in Women With HR+, HER2+ Locally Advanced or Metastatic Breast Cancer) are current studies in progress determining the response to management with trastuzumab and CDK4/6 inhibitors [23].

## Conclusions

There have been several effective therapies proposed for HER2 positive breast cancers in correlation with clinical trials. Discovering the mechanisms of trastuzumab resistance would increase its response to therapy and promote better clinical outcomes. Clinicians are being continuously challenged by the resistance mechanisms and bioavailability of the drugs in the treatment of metastatic breast cancers. The addition of new drugs to the chemotherapeutic regimen increases complexity, burden of side effects, and chances of relapse. Novel anti-HER2 agents have been directed towards therapy making a major paradigm shift.
